# Clinicopathological Findings on 28 Cases with XP11.2 Renal Cell Carcinoma

**DOI:** 10.1007/s12253-019-00792-0

**Published:** 2020-01-18

**Authors:** Levente Kuthi, Áron Somorácz, Tamás Micsik, Alex Jenei, Adrienn Hajdu, István Sejben, Dániel Imre, Boglárka Pósfai, Katalin Kóczián, Dávid Semjén, Zoltán Bajory, Janina Kulka, Béla Iványi

**Affiliations:** 1 Department of Pathology, University of Szeged, 1 Állomás Street, Szeged, H-6725 Hungary; 2 2nd Department of Pathology, Semmelweis University, Budapest, Hungary; 3 1st Department of Pathology and Experimental Cancer Research, Semmelweis University, Budapest, Hungary; 4 Department of Pathology, Bács-Kiskun County Teaching Hospital, Kecskemét, Hungary; 5 Department of Pathology, Hetényi Géza County Hospital, Szolnok, Hungary; 6 Department of Oncotherapy, University of Szeged, Szeged, Hungary; 7 Surgical and Molecular Tumor Pathology Centre, National Institute of Oncology, Budapest, Hungary; 8 Department of Pathology, Clinical Center and Medical School, University of Pécs, Pécs, Hungary; 9 Department of Urology, University of Szeged, Szeged, Hungary

**Keywords:** Translocation renal cell carcinoma, Xp11.2, Immunohistochemistry, *TFE3* gene, Fluorescence in situ hybridization (FISH)

## Abstract

Xp11.2 translocation carcinoma is a distinct subtype of renal cell carcinoma characterized by translocations involving the *TFE3* gene. Our study included the morphological, immunohistochemical and clinicopathological examination of 28 Xp11.2 RCCs. The immunophenotype has been assessed by using CA9, CK7, CD10, AMACR, MelanA, HMB45, Cathepsin K and TFE3 immunostainings. The diagnosis was confirmed by *TFE3* break-apart FISH in 25 cases. The ages of 13 male and 15 female patients, without underlying renal disease or having undergone chemotherapy ranged from 8 to 72. The mean size of the tumors was 78.5 mm. Forty-three percent of patients were diagnosed in the pT3/pT4 stage with distant metastasis in 6 cases. Histological appearance was branching-papillary composed of clear cells with voluminous cytoplasm in 13 and variable in 15 cases, including one tumor with anaplastic carcinoma and another with rhabdoid morphology. Three tumors were labeled with CA9, while CK7 was negative in all cases. Diffuse CD10 reaction was observed in 17 tumors and diffuse AMACR positivity was described in 14 tumors. The expression of melanocytic markers and Cathepsin K were seen only in 7 and 6 cases, respectively. TFE3 immunohistochemistry displayed a positive reaction in 26/28 samples. *TFE3* rearrangement was detected in all the analyzed cases (25/25), including one with the loss of the entire labeled break-point region. The follow-up time ranged from 2 to 300 months, with 7 cancer-related deaths. In summary, Xp11.2 carcinoma is an uncommon form of renal cell carcinoma with a variable histomorphology and rather aggressive clinical course.

## Introduction

In the current classification scheme there are 13 distinct types of renal cell carcinoma (RCC), and one of them is the Xp11.2 translocation RCC. It is a rare subtype and is characterized by different translocations involving the transcription factor 3 gene (*TFE3*), that leads to a new fusion gene encoding an aberrant transcription factor [1]. Five common partner genes were identified including *ASPL-TFE3*: t(X;17)(p11.2;q25), *PSF-TFE3*: t(X;1)(p11.2;p34), *PRCC-TFE3*: t(X;1)(p11.2;q21), *CLTC-TFE3*: t(X;17)(p11.2;q23) and *NonO-TFE3*: t(X)(p11.2q12) so far in the literature [2–5]. Although Xp11.2 RCC was described as a malignancy among children and adolescents, cases from adults and elders were also reported [6, 7]. The prognosis is controversial, since Xp11.2 RCC has an indolent behavior in children, however, new reports on an aggressive clinical course in adults has been reported as well [6, 8]. Tumor cells usually have blank cytoplasm that mimics clear cell RCC, although the growth pattern is frequently papillary, with psammoma bodies often present [9]. Xp11.2 RCC displays negativity with carbonic anhydrase 9 (CA9) and CK7 [10, 11], while CD10 is often positive and the expression of the melanocytic markers (MelanA and HMB45) are frequent, although they are not expressed in other subsets of RCC [6]. Cathepsin K is a novel marker for Xp11.2 RCC and its positivity indicates the presence of fusion gene *PRCC-TFE3* [12]. The result of translocations involving the *TFE3* gene is the overexpression of the TFE3 protein that can be detected by immunohistochemistry [13]. Although nuclear positivity of the TFE3 protein is a useful diagnostic marker, false negativity and positivity may occur, therefore the identification of the *TFE3* gene rearrangement by fluorescent in situ hybridization (FISH) is needed to confirm the diagnosis [13]. The prognosis of Xp11.2 RCC is still unclear because of the low appearance of series including a great number of patients and the short follow-up period [6]. The three main aims of this retrospective study were: (1) to determine the frequency of Xp11.2 RCC in a large set of surgically treated renal tumors; (2) to provide detailed survival data; and (3) to analyze the morphological features with immunohistochemical and genetic profile to help pathologists establish an accurate histological diagnosis.

## Material and Methods

### Case Selection

A retrospective study was performed that included morphological, immunohistochemical and molecular pathological analysis. The cases were collected from the Department of Pathology, University of Szeged (1512 own and 64 consultation cases), the 2nd Department of Pathology, Semmelweis University (818 cases), and the 1st Department of Pathology and Experimental Cancer Research, Semmelweis University (404 cases). The diagnostic criteria for Xp11.2 RCC were the typical morphological pattern or moderate-to-strong nuclear positivity with *TFE3* immunohistochemistry or a positive *TFE3* break-apart FISH analysis. A total of 28 cases of Xp11.2 RCC were diagnosed from 2804 tumors in the three centers. All tumors were selected for further immunohistochemical analysis. The main clinical data included age, sex and symptoms at the time of the diagnosis. Follow-up data were collected from the general practitioners, patient records as well as the patient database of the University of Szeged and Semmelweis University. Tumor size and AJCC TNM stage were obtained from the original histopathological report, however, the TNM stage was amended according to the eighth edition of AJCC TNM staging. All the hematoxylin-eosin stained slides were reviewed by three pathologists (LK, ÁS, TM) to reevaluate the grade according to the ISUP criteria, the histological pattern (generally papillary or solid pattern) and to estimate the percentage of the cellular morphology (predominantly clear or eosinophilic cells). The presence of foamy cells, intracellular pigment, cholesterol clefts, necrosis and psammoma bodies were also recorded, though the extent of necrosis was not scored.

### Immunohistochemistry (IHC) and Tissue Microarray (TMA)

The IHC reactions were carried out on TMA. The recipient TMA block was constructed by using a TMA Master (3DHISTECH Ltd., Budapest, Hungary). Stated briefly, from the most representative paraffin blocks of the tumors, two cylindrical cores of 2 mm in diameter were punched out manually. For IHC labeling a panel of antibodies listed in Table 1 was used. Only membrane labeling for CA9, and nuclear labeling for TFE3 was treated as positive. The scoring was performed in a semiquantitative manner and the cases were classified into three categories, namely negative (no staining or less than 5% of positive cells), focally positive (5–75% of positive cells) and diffusely positive (76–100% of positive cells).

**Table 1:** List of the antibodies used in the study

Antibody	Clonality/Source/Clone	Concentration
CA9	rabbit polyclonal, Novus Biologicals	1/2000
CK7	mouse monoclonal, Cell Marque, OV-TL 12/30	1/100
CD10	mouse monoclonal, Biocare Medical, CM129	1/50
AMACR	rabbit polyclonal, Abcam	1/100
MelanA	mouse monoclonal, Labvision, A103	1/200
HMB45	mouse monoclonal, Cell Marque, hmb-45	1/200
TFE3	rabbit monoclonal, Cell Marque, mrq-37	1/100
Cathepsin K	mouse monoclonal, Abcam, 3f9	1/100

### TFE3 Break-Apart FISH Analysis

Fluorescent in situ hybridization assays were carried out to detect *TFE3* gene rearrangement. Four μm thick sections were cut from the TMA blocks. The sections were deparaffinized and the reaction was carried out by using Zyto*Light*® SPEC *TFE3* dual color break-apart FISH probe (ZytoVision GMBH, Bremerhaven, Germany) according to the manufacturer’s instructions. The slides were counterstained with 4′, 6-diamidino-2-phenylindole (DAPI, Abbott Laboratories, Abbott Park, IL, USA) and scanned with a Pannoramic Midi slide scanner (3DHISTECH Ltd., Budapest, Hungary). The evaluation was performed by using Pannoramic Viewer (3DHISTECH Ltd., Budapest, Hungary). One hundred nuclei were counted and FISH reaction was considered positive when over 10% of the neoplastic nuclei displayed *TFE3* rearrangement.

## Results

Twenty-eight tumors proved to be Xp11.2 RCC among 2804 nephrectomies reviewed by the three pathology departments (0.99%). The diagnosis was suspected mainly because of the histological appearance. The diagnosis was later confirmed by IHC in each case and by FISH analysis except for *patient #11, #12* and *#24*.

### Clinical Data and Follow-Up

The clinicopathological findings are summarized in Table 2. Thirteen male and fifteen female patients were included in our cohort. The median age was 60 years (with range from 8 to 72). Three tumors occurred in children and Wilms’ tumor was suspected in all cases. The tumor produced symptoms in 9 patients; in *patient #22* a severe pain was provoked by distant bone metastasis. The tumor was an incidental finding in 7 patients. There were no underlying renal disorders in any patients in the affected kidney, but in *patient #12* contralateral kidney agenesis was present. None of the examined patients had received chemotherapy or had had previous malignant tumors, although pharyngeal carcinoma developed in *patient #2* after the nephrectomy.

**Table 2:** Clinicopathological features of the patients

	Age (y)	Sex	Symptoms†	Side	Size (mm)	pT Stageǁ	Node Status§	Metastasis or recurrence*	Follow-up (mo)	Status
1	52	M	–	L	70	4	–	–	–	LTF
2	69	M	Incidental finding on CT	L	50	1b	–	–	–	LTF
3	47	M	Hematuria, flank pain	R	100	3b	Neg	Lung, Liver	2	DOD
4	69	F	Hematuria	R	80	3a	–	Bone-U, Local R	53	DOD
5	59	M	Flank pain	L	140	4	Pos	–	–	LTF
6	67	M	Fatigue, subcostal pain	L	160	4	Pos	Liver, LN	2	DOD
7	40	M	–	L	25	1a	–	None	127	NED
8	15	F	Palpable ventral mass	L	55	1b	–	Lung, Vertebral column	14	DOD
9	46	M	Incidental finding	L	100	2a	Neg	Local R	13	DOD
10	72	F	Flank pain	L	140	3a	–	–	4	DOD
11	21	F	–	R	–	3a	–	–	–	LTF
12	14	M	–	L	–	1a	–	None	12	NCRD
13	31	M	Incidental finding on US	L	55	1b	–	None	87	NED
14	57	M	–	L	100	3a	Neg	Lung, Liver, Vertebral column	81	DOD
15	40	F	–	R	110	3a	–	LN	65	NED
16	50	F	–	R	45	1b	–	None	31	NED
17	32	M	Incidental finding	B	15	1a	–	None	24	NED
18	60	F	Incidental finding	–	16	1a	–	–	–	LTF
19	66	F	–	R	60	1b	Pos	Adrenal gland	–	LTF
20	32	M	–	R	20	1a	Pos	Liver	–	LTF
21	17	F	–	R	35	1a	–	None	175	NED
22	36	F	Shoulder pain	R	120	3b	–	Scapula	13	AWD
23	40	F	Incidental finding	R	41	1b	–	None	24	NED
24	8	F	Palpable ventral mass	L	100	2b	–	–	321	NED
25	54	F	–	R	120	3a	Neg	None	10	NED
26	66	M	Abdominal pain	L	110	2b	–	None	4	NED
27	51	F	–	R	65	1b	–	Vertebral column	3	AWD
28	46	F	Incidental finding	L	110	3a	Pos	None	7	NED

†Symptoms: including any tumor-related symptoms; incidental finding indicates a symptomless tumor; −, no data. ǁpT Stage: classification by AJCC 2016 TNM Staging System. §Node Status: nodal status at time of surgery; −, no lymph node was removed; Neg, negative; Pos, positive. A lymph node metastasis that developed during the follow-up period is listed in the” Metastasis” column. *Metastasis: either found earlier or at the same time with the primary renal tumor, or during the follow-up period; Bone-U, bone, exact location is unknown; −, no data; R, recurrence; LN, lymph node. ¶Status: DOD, died of disease; LTF, patient is deceased, but lost to follow-up; NED, no evidence of disease; NCRD, not a cancer-related death; AWD, alive with disease; −, no data

Radical nephrectomy was performed in each case except three patients, who were treated with nephron-sparing nephrectomy (tumor resection) because of the relatively small tumors (*patient #7* and *#8*) or the absence of the contralateral kidney (*patient #12*)*.*

Follow-up information was accessible in 21/28 patients and the mean follow-up time was 51 months (with range from 2 to 321 months). Regional lymph node or distant metastasis developed in 13 patients (9 had been discovered before surgery; 6 distant and 3 regional lymph node metastases). Seven patients died from cancer-related causes and one patient died from a non-cancer-related cause. In *patient #15* a regional lymph node metastasis developed after 12 months so she was treated with retroperitoneal lymphadenectomy and at the last follow-up there were no signs of the disease. However, 60 months after the nephrectomy, *patient #14* had multiple pulmonary, hepatic and bone metastases. He received tyrosine kinase and mTOR inhibitors until treatment failure. In *patient #27* multifocal vertebral metastases developed. She is currently receiving tyrosine kinase therapy and stable. The remaining 11 patients were alive with no evidence of disease.

### Morphological Aspects

**Fig. 1:**
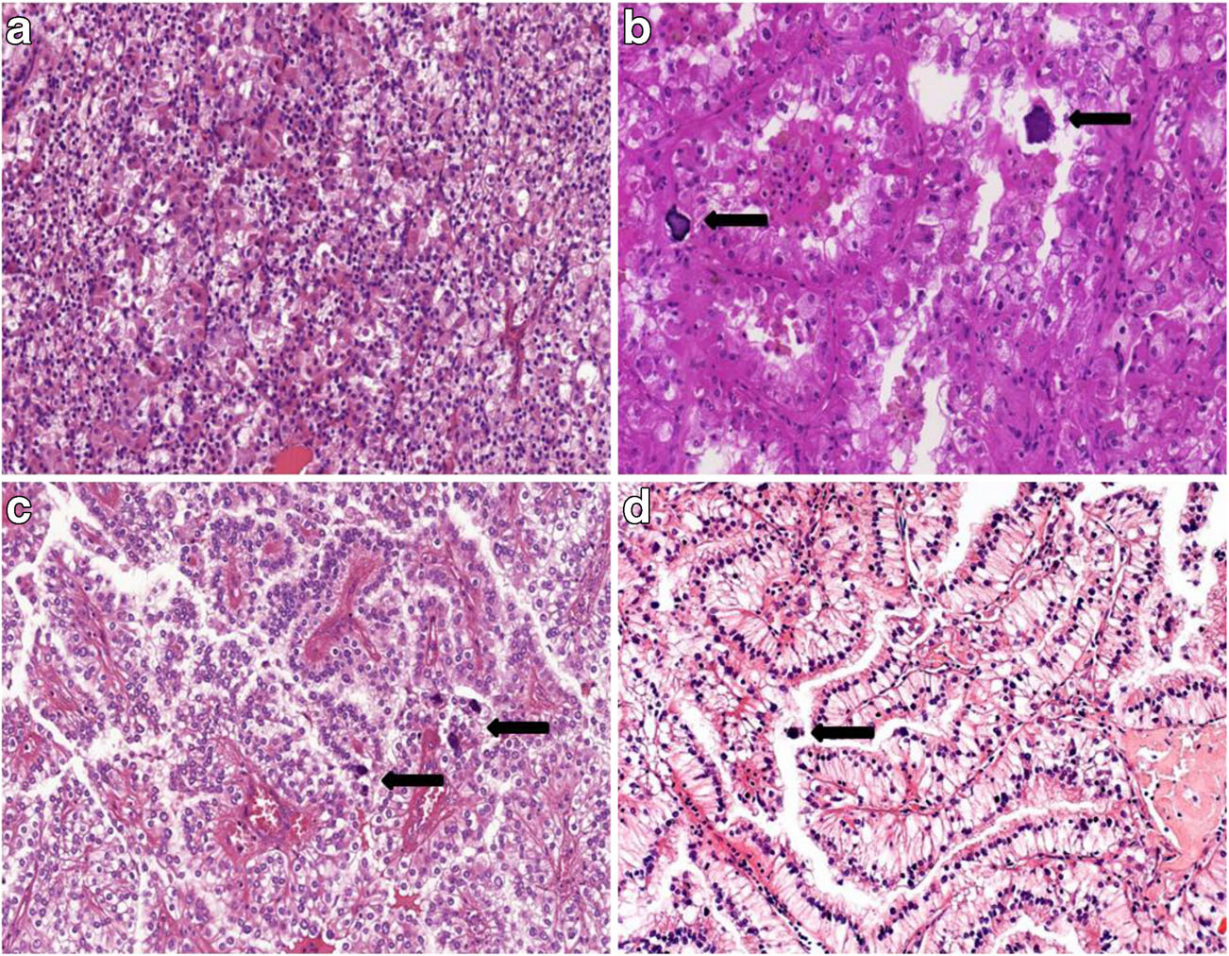
Representative images of typical morphological features of Xp11.2 renal cell carcinomas. (**A**) Solid-nested pattern with admixture of eosinophilic and clear cells. (**B)** Alveolar pattern populated by eosinophilic cells. Psammoma bodies are also present. (**C)** Papillary pattern with voluminous clear cells and psammoma bodies. **D** Occasionally the nuclei are near the apical surface of the cells and they mimic clear cell papillary renal cell carcinoma. The arrows indicate the psammoma bodies. All images have a magnification factor of 200x

**Fig. 2:**
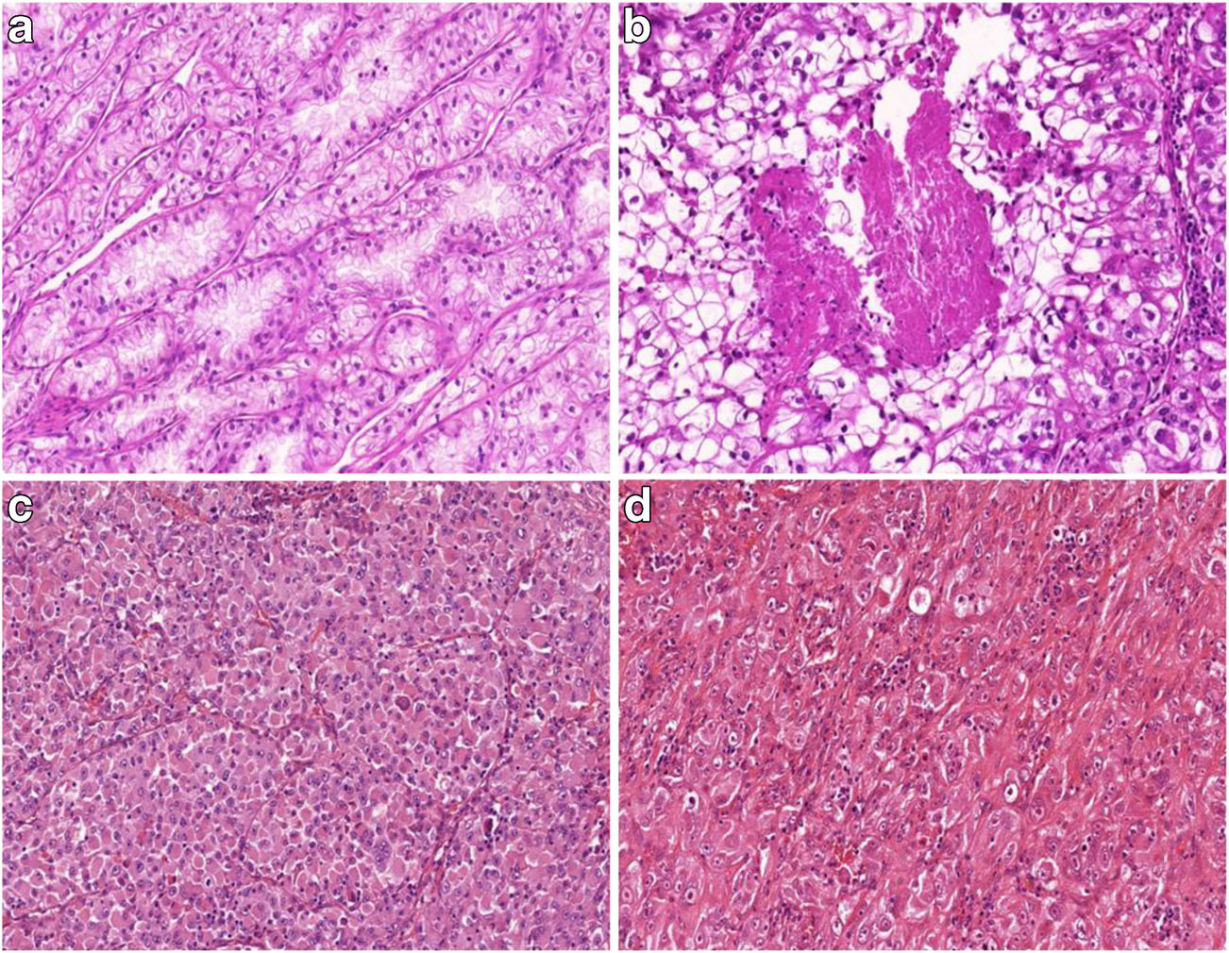
Representative images of Xp11.2 renal cell carcinomas with unusual morphological features. (**A**) Tubular pattern resembling low-grade clear cell carcinoma. **(B)** Solid pattern with foci of comedo-like necrosis. **(C)** Rhabdoid tumor-like pattern. **(D)** Anaplastic carcinoma appearance. All images have a magnification factor of 200x

All the examined tumors were unilateral and unifocal. The largest diameter of the tumors ranged from 15 mm to 160 mm and the average was 78.5 mm. In two cases the actual size of the tumor was unknown. Macroscopically the cut surface was usually solid and cystic with sulfur yellow color, as seen in clear cell RCC. Foci of necrosis or hemorrhage were occasionally noted as well. The invasion of the renal vein, sinus and adipose capsule was observed in 7, 8, 6 cases respectively. The predominant architectural appearance was solid pattern (13/28), followed by papillary pattern (11/28), while both solid and papillary patterns were seen in a small proportion of cases (4/28). Tumors were composed mostly of clear cells in 19 cases, mostly of eosinophilic cells in 7 cases and mixed clear cell and eosinophilic morphology was seen in 2 cases. Twenty-two cases had typical architecture with voluminous clear cytoplasm, nested or papillary growth. The remaining 6 cases had diverse architecture, mostly mimicking clear cell RCC, except for *patient* #5, whose tumor resembled rhabdoid morphology and *patient #6*, whose tumor had anaplastic carcinoma appearance. The presence of foamy cells, intracytoplasmic pigment, cholesterol clefts, psammoma bodies and necrosis were observed in 7, 4, 1, 11 and 17 cases respectively. Most of the tumors had high-grade nuclear features (19/28). Detailed summary of the microscopic findings can be found in Table 3. Figures 1 and 2 include representative images of the morphological features.

**Table 3:** Histological findings of the investigated tumors

	PP (%)	SP (%)	CCs (%)	ECs (%)	Foamy cells	IP	ChC	PB	Necrosis	ISUP grade
1	5	95	50	50	–	–	–	–	+	4
2	40	60	30	70	+	+	–	–	–	2
3	1	99	20	80	–	–	–	–	+	4
4	50	50	90	10	–	–	–	–	+	2
5	–	100	10	90	–	–	–	–	+	4
6	–	100	5	95	–	–	–	–	+	4
7	–	100	100	–	–	–	–	–	–	1
8	90	10	80	20	–	–	–	–	–	2
9	50	50	70	30	–	–	–	–	+	4
10	80	20	75	25	–	–	–	–	+	4
11	95	5	90	10	+	–	–	–	–	3
12	50	50	80	20	–	–	–	+	+	3
13	100	–	100	–	+	–	+	+	+	2
14	10	90	60	40	–	+	–	+	–	2
15	80	20	70	30	–	+	–	–	+	3
16	90	10	75	25	+	–	–	+	–	3
17	100	–	100	–	–	–	–	+	–	2
18	5	95	30	70	+	–	–	+	–	3
19	10	90	80	20	–	–	–	+	+	2
20	–	100	95	5	–	–	–	+	+	4
21	–	100	30	70	–	–	–	+	–	2
22	50	50	90	10	–	–	–	–	+	3
23	–	100	80	20	+	–	–	–	+	3
24	–	100	60	40	–	–	–	–	–	3
25	80	20	30	70	+	–	–	+	+	3
26	95	5	90	10	–	+	–	–	+	3
27	100	–	50	50	–	–	–	+	–	3
28	90	10	80	20	–	–	–	–	+	3

PP, indicates papillary pattern; SP, solid pattern; CCs, clear cells; ECs, eosinophilic cells; IP, intracytoplasmic pigment; ChC, cholesterol clefts; PB, psammoma bodies; +, present; −, absent

### IHC Findings

**Fig. 3:**
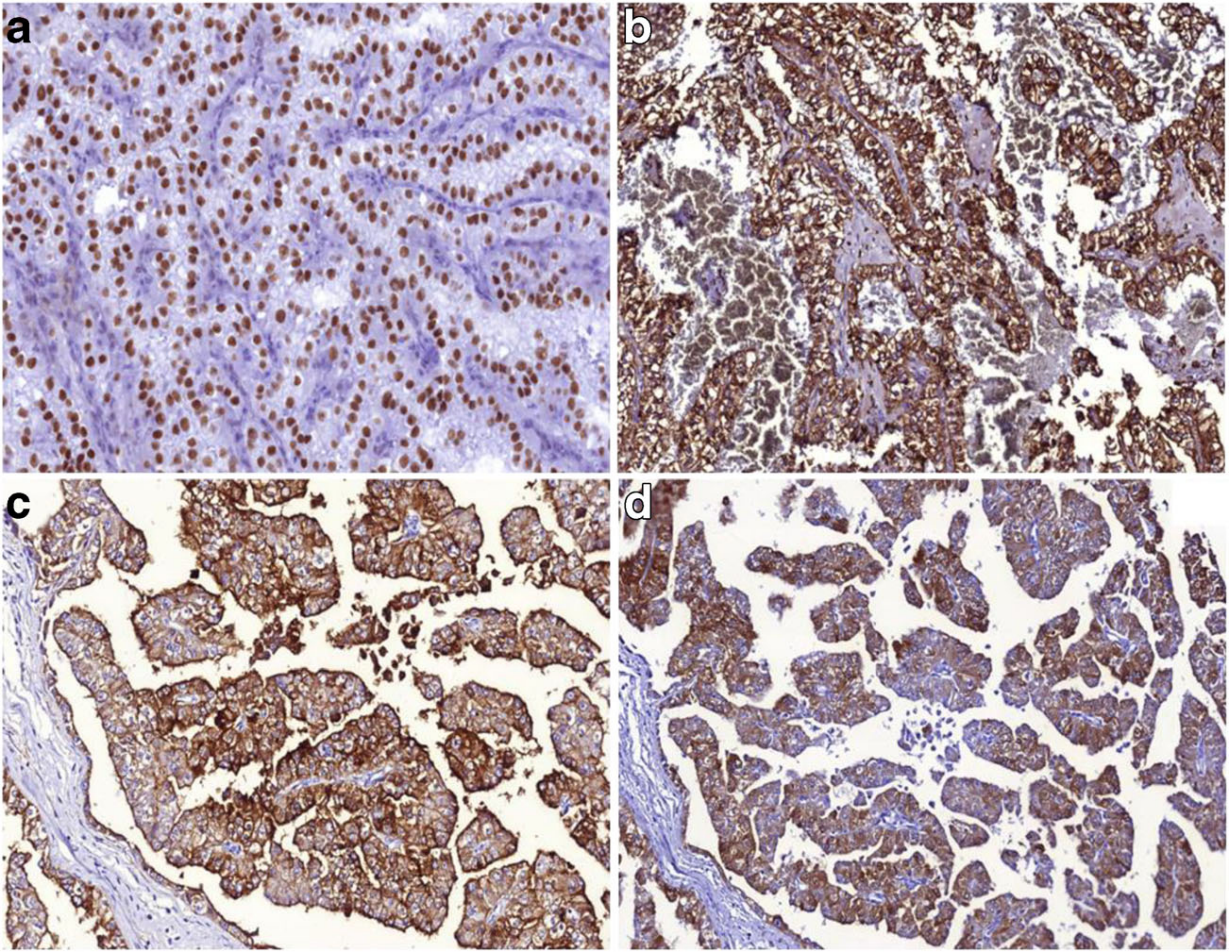
Representative images of the immohistochemical features of the analyzed tumors. (**A**) Tumor cells display diffuse TFE3 nuclear positivity. (**B)** Cathepsin K expression in an Xp11.2 renal cell carcinoma. **(C)** Diffuse cytoplasmic and membranous CD10-positivity is frequently seen in Xp11.2 renal cell carcinomas. **(D)** MelanA expression in Xp11.2 renal cell carcinoma. All images have a magnification factor of 200x

Results of immunohistochemistry are summarized in Table 4 and representative pictures are presented on Fig. 3. Three cases displayed positivity with CA9, although two of these were necrotic tumors. All the examined cases were negative with CK7, while CD10 was strongly positive in 17 cases. AMACR was negative in 14 tumors and a diffuse-to-focal positivity was seen in the remaining 14 cases. The diagnostic *TFE3* reaction strongly labeled the nuclei in 26/28 cases, however, Cathepsin K displayed positivity only in 6 tumors. MelanA was positive in four cases and HMB45 showed a weak-to-diffuse positivity in three patients.

**Table 4:** Immunohistochemical results of the analyzed cases

	CA9	CK7	CD10	AMACR	MelanA	HMB45	Cathepsin K	TFE3 IHC	TFE3 FISH
1	N	N	D	N	N	N	N	D	+
2	N	N	D	N	N	N	N	D	+
3	N	N	N	N	N	N	N	D	+
4	N	N	D	D	N	N	D	D	+
5	N	N	D	N	N	N	N	D	+
6	N	N	N	N	N	N	N	D	+
7	N	N	D	F	N	N	N	D	+
8	N	N	D	D	N	N	N	D	+
9	N	N	N	N	N	N	N	D	+
10	N	N	D	N	D	N	N	D	+
11	N	N	D	F	N	N	F	F	NA
12	N	N	D	D	N	N	N	D	NA
13	N	N	D	D	N	D	N	D	+
14	N	N	D	D	N	F	D	D	+
15	N	N	N	N	D	N	N	D	+
16	N	N	D	D	N	N	N	D	+
17	N	N	D	F	N	N	N	D	+
18	N	N	F	D	N	N	N	F	+
19	N	N	F	N	N	N	N	D	+
20	F	N	N	N	N	N	N	N	+
21	N	N	D	N	N	D	D	D	+
22	N	N	F	D	N	N	F	D	+
23	N	N	N	N	N	N	N	D	+
24	N	N	N	N	D	N	N	F	NA
25	N	N	D	D	N	N	N	F	+
26	N	N	D	D	F	N	F	F	+
27	F	N	D	N	N	N	N	N	+
28	F	N	F	F	N	N	N	D	+

N, indicates negative; F, focally positive; D, diffusely positive; NA, data not available

### FISH Findings

**Fig. 4:**
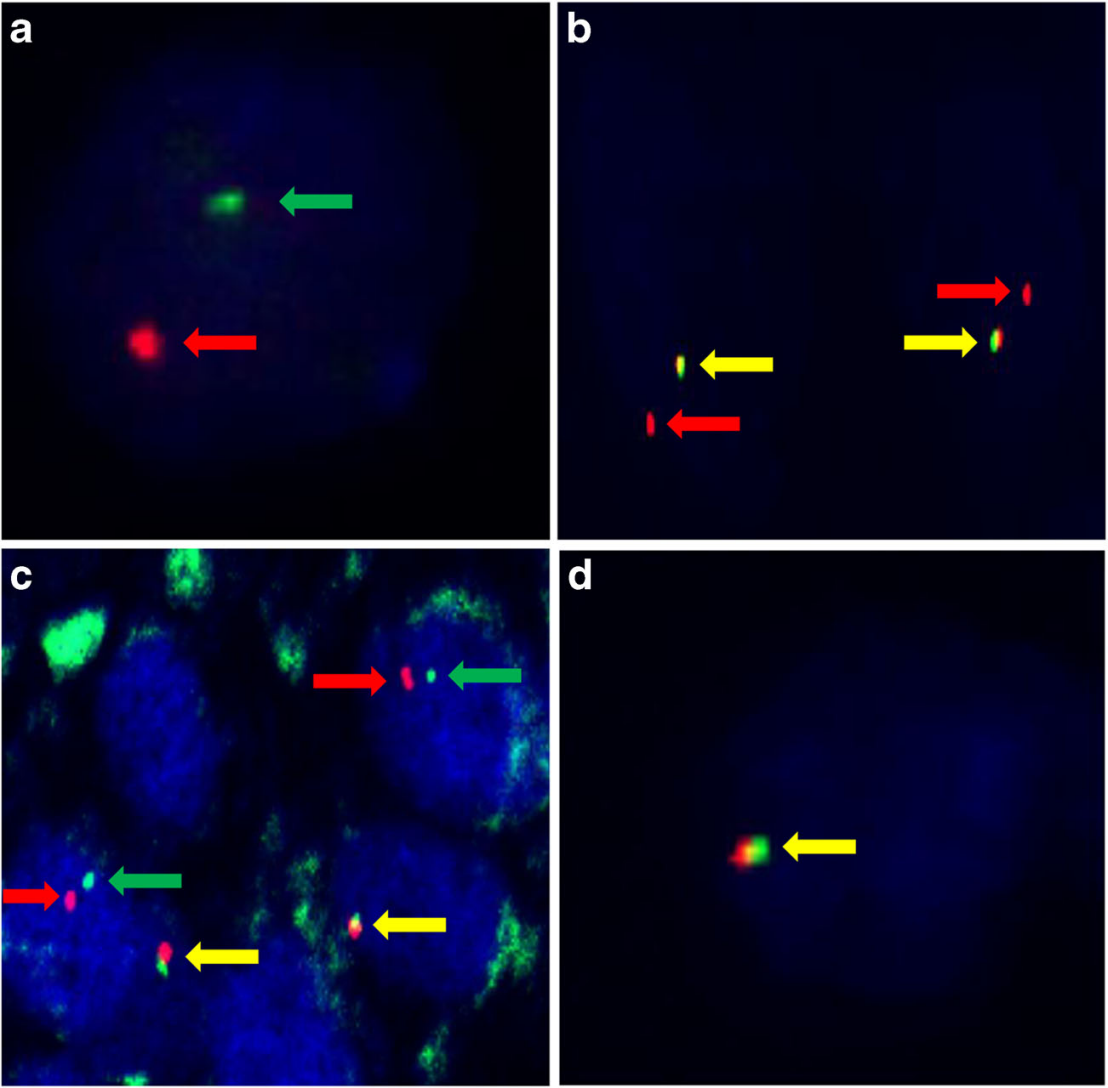
Representative images of the signal patterns seen in the analyzed tumors. (**A**) Typical split signals (red and green arrows) are present in a male patient (*patient #17)*. **(B)** Truncated signal pattern consisting of a pair of fused signals (yellow arrows) and a single red signal was observed in *patient #10*. **(C)** Although signals are separated (red and green arrows), they are unusually close to each other. In lymphocytes normal fused signals (yellow arrows) are present (*patient #14*). **(D)** The loss of an entire break-point region was observed in *patient #23*. The yellow arrow indicates the intact chromosome X

FISH reaction was performed in 25 cases, because in *patient #11*, *#12* and *#24*, the quality of tumor tissue was not appropriate for proper molecular analysis. For the above-mentioned three cases, FISH was repeated using the original paraffin blocks, although the test remained unsuccessful. In 21 tumors typical split signals were seen (Fig. 4 A), while in *patient #9* and *#10* truncated signal pattern was mostly observed (Fig. 4 B). In *patient #14*, signals were separated, even though they were unusually close to each other (Fig. 4 C). In *patient #23* (a female) an entire break-point region was completely absent (Fig. 4 D). Hence in this case only one signal pair was detected in the nuclei of the tumor cells, while in the surrounding renal parenchyma two unaffected signal pairs were present. The immunophenotype and histomorphology led us to classify the case as Xp11.2 RCC. No other abnormalities were seen by using FISH in any cases.

## Discussion

### Clinicopathological Findings of Xp11.2 RCC

We reviewed 2804 nephrectomy cases and identified 28 Xp11.2 translocation RCCs. In our cohort its frequency was lower, compared to literature data (0.99 vs. 1–4%) [22]. That is due to the relatively large number of patients included in our study, the analysis may roughly represent the ratio of Xp11.2 RCC in Hungary. In these neoplasms the characteristic cytogenetic change is the translocation involving the MIT family transcription factor *TFE3* gene [1]. These tumors were once regarded as childhood malignancies, though because of the significant overlapping features with clear cell and papillary RCC and the limited cytogenetic data, some authors suggested that the exact frequency in adults is underestimated [14]. In our cohort 12 tumors occurred in patients who were less than 40 years old, only four tumors affected children, and the oldest patient examined was 72 years old. Nine patients were symptomatic and tumor-related pain was the most common symptom. Only seven tumors did not produce any noticeable signs. A link between previous use of chemotherapy and translocation RCC was reported [15], but in our study there was no patient with a prior history of malignancy. Discriminating Xp11.2 RCC from other subtypes of RCC is crucial for prognostic and predictive reasons. It was suggested recently, that patients with Xp11.2 RCC may benefit from mTOR inhibitors and VEGF-targeted agents [16, 17]. The diagnosis relies on morphological features, immunohistochemical findings and molecular pathological analysis. Xp11.2 RCC has no specific macroscopic appearance; in fact most tumors resemble clear cell RCC with a sulfur yellow cut surface along with foci of hemorrhage and/or necrosis [9]. Xp11.2 RCC is usually diagnosed as a sizeable mass in the kidney. The mean size of our tumors (78.5 mm) was larger than in the earlier reported series [2, 14, 18]. In our previous analysis, only unclassified RCC and collecting duct carcinoma were larger than Xp11.2 RCC [19]. An invasion of the renal vein and/or the sinus is quite frequent; at least one of these was noticed in 12 patients. Metastatic spread to the regional lymph nodes or distant organs was observed in 32% of the cases; six patients had nephrectomy at the pM1 stage. Our observations on the rate of pT3/pT4 stage and the occurrence of metastasis are in accordance with the literature data [14]. This late stage discovery might partly explain the generally poor outcome in Xp11.2 RCC.

### Microscopic Features of Xp11.2 RCC

Microscopically the predominant growth patterns are papillary, tubular, nested and mixed. A striking histological finding is the presence of psammoma bodies [2, 18]. A different distribution was observed in our cohort, as a result of solid pattern observed as most frequent, followed by papillary and mixed architecture. Tumors were composed of mainly clear cells in 19 and of eosinophilic cells in 7 cases. Additionally the simultaneous presence of both cell types was noted in two cases. Psammoma bodies were observed in 11 cases. Foamy cells and intracytoplasmic pigment are common features in papillary RCC; hence their extensive presence can cause differential diagnostic problems. However, in our cohort both foamy cells and intracytoplasmic pigment occurred in a small proportion of cases and they did not have a predominant papillary pattern. Microscopic tumor cell necrosis was observed in 60% of tumors. Although the effect of necrosis on the outcome in Xp11.2 RCC is doubtful, in a large set of RCC patients, poor prognostic effect of microscopic tumor necrosis was identified earlier in the three most frequent types of RCC [20]. Cases with atypical architecture can cause serious diagnostic difficulties; namely, the morphological spectrum of Xp11.2 RCC is quite broad, furthermore urothelial cell carcinoma mimicking translocation RCC was reported as well [13]. In our cohort, a case mimicking anaplastic carcinoma and another with rhabdoid morphology were observed. Some authors suggested that the specific translocation has an influence on histological appearance [21]. Because of the absence of fusion partner analysis, we cannot argue for or against this statement. The influence on the prognosis of the fusion partner is yet unclear [23].

### Immunophenotype of Xp11.2 RCC

Xp11.2 RCC is negative with CA9, CK7 and positive with CD10 and AMACR [10]. In our series, both CA9 and CK7 were completely negative in almost every case, except for CA9 in three samples. However, two of these tumors were extensively necrotic, therefore we concluded that the staining was related to hypoxia of the tumor tissue. Diffuse CD10 labeling was noted in 60% of cases, while AMACR-positivity was observed only in 50% of the tumors. MelanA and HMB45 expression is frequent in *TFEB* translocation RCC, nevertheless is rare in Xp11.2 translocation RCC [5]. Similar proportion was described in our cohort; namely, MelanA and HMB45 were positive in 4 and 3 tumors respectively. For the diagnosis of Xp11.2 RCC, TFE3 immunostaining is the most frequently used method. The specificity and sensitivity of the immunohistochemistry were found to be 99.6% and 97.5% [24], although in some cases false negativity and false positivity can occur [23, 25]. Argani et al. described that the shorter incubation time with automated detection system made TFE3 IHC more sensitive, while the specificity of the reaction decreased. For the false-negative results, some authors declared that it can be caused by preanalytical factors (e.g. fixation time) or by different analytical methodologies applied (e.g. poor-quality antibodies and inappropriate antigen retrieval) [13, 26]. There were only two tumors (*patient #20* and *#27*) with *TFE3*-negativity and the remaining cases displayed a diffuse and strong nuclear positivity. Therefore, in our analysis the sensitivity of TFE3 immunohistochemistry correlates with data of the earlier reports [24]. Cystine protease Cathepsin K is a novel immunohistochemical marker for Xp11.2 RCC, although the expression depends on the fusion partner of the *TFE3*. This can serve as an explanation of its expression in only approximately 60% of Xp11.2 RCC [12, 27]. Six cases with Cathepsin K-positivity were described, that is slightly under the reported rate in literature [13]. The difference might be related to the different translocation partners.

### Genetic Markers of Xp11.2 RCC

Reverse transcription polymerase chain reaction (RT-PCR) is a sensitive method for the identification of different kinds of chimeric mRNA transcripts, though the limited availability of frozen samples generally makes the testing problematic or even impossible [28]. Cytogenetic karotyping is another classic methodology for recognizing structural changes among chromosomes. Nevertheless it requires a special laboratory, technicians and fresh material, hence the use of karotyping in the routine diagnosis of solid tumors is limited. Currently next-generation sequencing can be used to reveal the partner genes and identify new ones, as Pei et al. did [29]. FISH reactions have been performed on FFPE samples with satisfactory for some time now. *TFE3* break-apart FISH assay was introduced in 2011 and since that it has become an indispensable diagnostic tool for Xp11.2 RCC [13, 30]. In our study the diagnosis was supported with *TFE3* break-apart FISH analysis in 25 cases. The classic break-apart pattern was observed in 21 tumors and the truncated signal pattern was noted in 2 cases. For the latter cases, two explanations exist. First of all, truncation effect of cutting the tumor cell nuclei is a well-known problem in FISH assays on FFPE slides [13]. On the other hand, in our cases this was the dominant pattern. In *patient #9* a single red signal was seen in 100% of the positive tumor cells, while in *patient #10* split and a single red signal was observed in 17% and 83% of the positive tumor cells respectively. Therefore it is considered by our team that the labeled part of the *TFE3* was lost due to an atypical break in the gene sequence. Atypical FISH patterns are known for both epitheloid renal neoplasia along with soft tissue sarcomas [30–32]. In *patient #14* the signals were unusually close to each other, and this phenomenon is the indicative of intrachromosomal inversion [4]. In a female patient an entire break-point region was completely missing from the majority of the tumor cells. This was considered to be a result of an atypical translocation. Otherwise, the histomorphology and immunophenotype were concordant with Xp11.2 RCC, so the final diagnosis was made on summary of the above-mentioned results. Such signal pattern was presumed earlier [30], though to our best knowledge, this is the first report of such a signal pattern in Xp11.2 RCC. Optimized *TFE3* break-apart FISH assay is extremely useful in routine diagnosis and pathology consultation [27]. However, FISH analysis has its own limitations as well, whereas poor fixation, inappropriate hybridization, and/or extensive contamination with normal stromal cells can lead to negative results. It must be stated that break-apart FISH test has low sensitivity for intrachromosomal or paracentric inversions like in *RBM10-TFE3* and *NonO-TFE3* RCCs [33]. In these cases, despite the typical microscopic appearance and the characteristic immunophenotype, FISH can provide equivocal or even negative results. In this particular scenario, one must be really cautious about setting the diagnosis as Xp11.2 RCC, and fusion partner analysis (if available) by RT-PCR or RNA sequencing should be considered [33].

### Clinical Course of Xp11.2 RCC

Some authors previously reported that Xp11.2 RCC had an indolent course [35–38]. Camparo et al. calculated a mortality rate of 13.6% for Xp11.2 RCC from their analysis and literature data, although the follow-up period was quite short [14]. A fascinating case of Xp11.2 RCC was reported by Mangel et al. [38]. In their report, rapid progression of a stable disease was noted after the patient became pregnant, and the authors considered that the enormous tumor evolution was triggered by cytokines and hormones produced by the placenta, especially human chorionic gonadotropin. In our cohort the median follow-up time was 14 months. In the meantime, 33% of the patients died from a cancer-related cause. This indicates the fact that Xp11.2 RCC has the same mortality rate as the calculated rate for overall RCC patients [39].

The strength of our study is the relatively high number of systematically analyzed cases by descriptive light-microscopy, a panel of immunohistochemistry and FISH analysis. Two tumors with a fairly unusual morphology was included, one with anaplastic carcinoma appearance and another with rhabdoid morphology. Unique FISH pattern with the complete loss of the labeled break-point region was observed. The clinical follow-up was not complete for all patients, however, the mean follow-up period was more than 4 years.

One limitation of this current study is the absence of cytogenetic studies and the data for fusion partner analysis.

In summary, the results of 28 Xp11.2 RCC cases were presented from a large surgically treated series of RCC. Xp11.2 RCC is a rare form of renal cell carcinoma; and it is accounted for 0.99% of all RCC cases in our study. In adults the outcome is rather poor. Cases with an unusual histomorphology may cause differential diagnostic problems, though the use of antibodies in combination can improve the diagnostic performance. Finally, to avoid false negative and false positive cases, the use of *TFE3* break-apart FISH studies and/or fusion partner analysis are strongly recommended.
